# Supplementary material for: Patterns of healthcare services utilization associated with intimate partner violence (IPV): Effects of IPV screening and receiving information on support services in a cohort of perinatal women
Source: PLoS One. 2020 Jan 31;15(1):e0228088. doi: 10.1371/journal.pone.0228088 (PMC6994004; doi:10.1371/journal.pone.0228088)
Supplement: S1 Appendix — (DOCX) [file pone.0228088.s001.docx]

**Appendix 1 – Associations between women’s characteristics and healthcare utilization measures at first-year follow-up in the total sample and the study groups (Arab and Jewish women)**

|  | **Total (%)** | **Family physician** | | **Gynecologist** | | **Specialist** | | | **ER** | | | **Hospitalization** | | | |
| --- | --- | --- | --- | --- | --- | --- | --- | --- | --- | --- | --- | --- | --- | --- | --- |
|  |  | **High use** | **P** | **Yes** | **P** | **Yes** | **P** | | **Yes** | **P** | | **Yes** | **P** | |  |
|  | **A1a- Total Sample (N=869 )** | | | | | | | | | | | | | | |
| **Age** |  |  |  |  |  |  |  | |  |  | |  |  | |  |
| 16-24 | 174 (20.0) | 88 (50.6) | 0.434 | 128 (73.6) | 0.903 | 68 (39.1) | **0.019** | | 74 (42.5) | **0.002** | | 22 (12.6) | 0.174 | |  |
| 25-34 | 520 (59.8) | 290 (55.8) |  | 390 (75.0) |  | 261 (50.2) |  |  | 155 (29.8) |  |  | 43 (8.3) |  |  |  |
| 35-48 | 175 (20.1) | 99 (56.6) |  | 129 (73.7) |  | 92 (52.6) |  |  | 46 (26.3) |  |  | 20 (11.4) |  |  |  |
| **Marital status** |  |  |  |  |  |  |  | |  |  | |  |  | |  |
| Married | 832 (96.1) | 451 (54.2) | **0.085** | 619 (74.4) | 0.552 | 405 (48.7) | 0.248 | | 262 (31.5) | 0.524 | | 82 (9.9) | 0.567 | |  |
| Other | 34 (3.9) | 23 (67.6) |  | 25 (73.5) |  | 14 (41.2) |  |  | 11 (32.4) |  |  | 3 (8.8) |  |  |  |
| **Education (woman)** |  |  |  |  |  |  |  | |  |  | |  |  | |  |
| High school or less | 375 (43.2) | 184 (49.1) | 0.109 | 284 (75.7) | 0.727 | 162 (43.2) | **0.020** | | 134 (35.7) | **0.016** | | 51 (13.6) | **0.001** | |  |
| Postsecondary or college | 139 (16.0) | 56 (40.3) |  | 101 (72.7) |  | 69 (49.6) |  |  | 48 (34.5) |  |  | 17 (12.2) |  |  |  |
| Bachelor or above | 355 (40.9) | 152 (42.8) |  | 262 (73.8) |  | 190 (53.5) |  |  | 93 (26.2) |  |  | 17 (4.8) |  |  |  |
| **Pregnancy status** |  |  |  |  |  |  |  | |  |  | |  |  | |  |
| Pregnant | 202 (23.4) | 118 (58.4) | 0.140 | 190 (94.1) | **0.001** | 77 (38.1) | **0.001** | | 109 (54.0) | **0.001** | | 39 (23.3) | **0.001** | |  |
| After birth | 662 (76.6) | 356 (53.8) |  | 453 (68.4) |  | 341 (51.5) |  |  | 164 (24.8) |  |  | 44 (6.6) |  |  |  |
| **Mother of children** |  |  |  |  |  |  |  | |  |  | |  |  | |  |
| Yes | 808 (93.1) | 370 (45.8) | 0.108 | 588 (72.8) | **0.001** | 399 (49.4) | **0.021** | | 234 (29.0) | **0.001** | | 73 (9.0) | **0.01** | |  |
| No | 60 (6.9) | 38 (63.3) |  | 58 (96.7) |  | 21 (35.0) |  |  | 40 (66.7) |  |  | 12 (20.0) |  |  |  |
| **Chronic disease** |  |  |  |  |  |  |  | |  |  | |  |  | |  |
| Yes | 83 (10.3) | 59 (71.1) | **0.002** | 61 (73.5) | 0.896 | 47 (56.6) | 0.165 | | 26 (31.3) | 1.000 | | 7 (8.4) | 0.846 | |  |
| No | 719 (89.7) | 408 (53.1) |  | 570 (74.1) |  | 369 (48.0) |  |  | 241 (31.3) |  |  | 77 (10.0) |  |  |  |
|  | **A2b- Jewish women (N=542)** | | | | | | | | | | | | | | |
| **Age** |  |  |  |  |  |  | |  |  | |  |  |  | |  |
| 16-24 | 43 (7.9) | 23 (53.5) | 0.922 | 30 (69.8) | 0.579 | 23 (53.5) | | 0.976 | 19 (44.4) | | 0.054 | 3 (7.0) | 0.857 | |  |
| 25-34 | 360 (66.4) | 202 (56.1) |  | 273 (75.8) |  | 193 (53.6) | |  | 103 (28.6) | |  | 26 (7.2) |  |  |  |
| 35-48 | 139 (25.6) | 76 (54.7) |  | 101 (72.7) |  | 73 (52.5) | |  | 35 (25.2) | |  | 12 (8.6) |  |  |  |
| **Marital status** |  |  |  |  |  |  | |  |  | |  |  |  | |  |
| Married | 508 (94.2) | 277 (54.5) | 0.151 | 379 (74.9) | 0.652 | 274 (53.9) | | 0.194 | 145 (28.5) | | 0.657 | 38 (7.5) | 0.654 | |  |
| Other | 31 (5.8) | 21 (67.7) |  | 22 (71.0) |  | 13 (41.9) | |  | 10 (32.3) | |  | 3 (9.7) |  |  |  |
| **Education (woman)** |  |  |  |  |  |  | |  |  | |  |  |  | |  |
| High school or less | 171 (31.5) | 86 (50.3) | 0.182 | 130 (76.0) | 0.583 | 79 (46.2) | | **0.043** | 60 (35.1) | | 0.055 | 19 (11.1) | 0.052 | |  |
| Postsecondary or college | 91 (16.8) | 56 (61.5) |  | 64 (70.3) |  | 47 (51.6) | |  | 28 (30.8) | |  | 8 (8.8) |  |  |  |
| Bachelor's degree or above | 280 (51.7) | 159 (56.8) |  | 210 (75.0) |  | 163 (58.2) | |  | 69 (24.6) | |  | 14 (5.0) |  |  |  |
| **Pregnancy status** |  |  |  |  |  |  | |  |  | |  |  |  | |  |
| Pregnant | 79 (14.6) | 53 (67.1) | **0.028** | 74 (93.7) | **0.001** | 36 (45.6) | | **0.144** | 47 (59.5) | | **0.001** | 14 (17.7) | **0.001** | |  |
| After birth | 461 (85.4) | 248 (53.8) |  | 328 (71.1) |  | 251 (54.4) | |  | 110 (23.9) | |  | 27 (5.9) |  |  |  |
| **Mother of children** |  |  |  |  |  |  | |  |  | |  |  |  | |  |
| yes | 520 (96.1) | 288 (55.4) | 0.874 | 383 (73.7) | **0.026** | 282 (54.2) | | **0.021** | 142 (27.3) | | **0.001** | 37 (7.1) | **0.043** | |  |
| no | 21 (3.9) | 12 (57.1) |  | 20 (95.2) |  | 6 (28.6) | |  | 14 (66.7) | |  | 4 (19.0) |  |  |  |
| **Chronic disease** |  |  |  |  |  |  | |  |  | |  |  |  | |  |
| yes | 76 (14.5) | 54 (71.1) | **0.003** | 57 (75.0) | 0.814 | 44 (57.9) | | 0.472 | 22 (28.9) | | 0.502 | 6 (7.9) | 0.922 | |  |
| no | 449 (85.5) | 237 (52.8) |  | 331 (73.7) |  | 240 (53.5) | |  | 127 (28.3) | |  | 34 (7.6) |  |  |  |
|  | **A1c- Arab women (N = 327)** | | | | | | | | | | | | |  |  |
| **Age** |  |  |  |  |  |  |  | |  |  | |  |  | |  |
| 16-24 | 131 (40.1) | 65 (49.6) | 0.288 | 98 (74.8) | 0.579 | 45 (34.4) | 0.102 | | 55 (42.0) | 0.188 | | 19 (14.5) | 0.165 | |  |
| 25-34 | 160 (48.9) | 88 (55.0) |  | 117 (73.1) |  | 68 (42.5) |  |  | 52 (32.5) |  |  | 17 (10.6) |  |  |  |
| 35-48 | 36 (11.0) | 23 (63.9) |  | 28 (77.8) |  | 19 (52.8) |  |  | 11 (30.6) |  |  | 8 (22.2) |  |  |  |
| **Marital status** |  |  |  |  |  |  |  | |  |  | |  |  | |  |
| Married | 324 (99.1) | 174(53.7) | 0.557 | 240 (74.1) | 0.409 | 131 (40.4) | 0.643 | | 117(36.1) | 0.704 | | 44 (13.6) | 0.647 | |  |
| Other | 3 (0.9) | 2 (66.7) |  | 3 (100.0) |  | 1 (33.3) |  |  | 1 (33.3) |  |  | 0 (0.0) |  |  |  |
| **Education (woman)** |  |  |  |  |  |  |  | |  |  | |  |  | |  |
| High school or less | 204 (62.4) | 105(51.5) | 0.528 | 154 (75.5) | 0.518 | 83 (40.7) | 0.549 | | 74 (36.6) | 0.550 | | 32 (15.7) | **0.020** | |  |
| Postsecondary or college | 48 (14.7) | 27 (56.3) |  | 37 (77.1) |  | 22 (45.8) |  |  | 20 (41.7) |  |  | 9 (18.8) |  |  |  |
| Bachelor's degree or above | 75 (22.9) | 44 (58.7) |  | 52 (69.3) |  | 27 (36.0) |  |  | 24 (32.0) |  |  | 3 (4.0) |  |  |  |
| **Pregnancy status** |  |  |  |  |  |  |  | |  |  | |  |  | |  |
| Pregnant | 123 (38.0) | 65 (52.8) | 0.484 | 116 (94.3) | **0.001** | 41 (33.3) | **0.027** | | 62 (50.4) | **0.001** | | 25 (20.3) | **0.002** | |  |
| After birth | 201 (62.0) | 108 53.7) |  | 125 (62.2) |  | 90 (44.8) |  |  | 54 (26.9) |  |  | 17 (8.5) |  |  |  |
| **Mother of children** |  |  |  |  |  |  |  | |  |  | |  |  | |  |
| yes | 288 (88.1) | 150 (52.1) | **0.060** | 205 (71.2) | **0.001** | 117 (40.6) | 0.470 | | 92 (31.9) | **0.001** | | 36 (12.5) | 0.132 | |  |
| no | 39 (11.9) | 26 (66.7) |  | 38 (97.4) |  | 15 (38.5) |  |  | 26 (66.7) |  |  | 8 (20.5) |  |  |  |
| **Chronic disease** |  |  |  |  |  |  |  | |  |  | |  |  | |  |
| yes | 7 (2.5) | 5 (71.4) | 0.345 | 4 (57.1) | 0.379 | 3 (42.9) | 0.892 | | 4 (57.1) | 0.241 | | 1 (14.3) | 0.948 | |  |
| no | 320 (97.5) | 171 (53.4) |  | 239 (74.7) |  | 129 (40.3) |  |  | 114 (35.6) |  |  | 43 (13.4) |  |  |  |
