# Supplementary material for: Patterns of healthcare services utilization associated with intimate partner violence (IPV): Effects of IPV screening and receiving information on support services in a cohort of perinatal women
Source: PLoS One. 2020 Jan 31;15(1):e0228088. doi: 10.1371/journal.pone.0228088 (PMC6994004; doi:10.1371/journal.pone.0228088)
Supplement: S2 Appendix — (DOCX) [file pone.0228088.s002.docx]

**Appendix 2- Summary of univariate associations^b^ between IPV variables, IPV screening,**

**information received, and both, and HCS utilization variables^a^**

|  | **Total sample** | | | | |  | **Jewish women** | | | | |  | **Arab women** | | | | |
| --- | --- | --- | --- | --- | --- | --- | --- | --- | --- | --- | --- | --- | --- | --- | --- | --- | --- |
|  | **community** | | | **hospital** | |  | **community** | | | **hospital** | |  | **community** | | | **hospital** | |
|  | **1** | **2** | **3** | **4** | **5** |  | **1** | **2** | **3** | **4** | **5** |  | **1** | **2** | **3** | **4** | **5** |
| **Any IPV** |  | **P** | **N** |  | **P** |  |  |  |  | **N** |  |  |  | **P** |  | **P** | **P** |
| **Physical IPV** | **N** |  |  |  |  |  | **N** |  |  | **N** |  |  |  |  |  |  |  |
| **Emotional IPV** | **N** | **P** | **N** |  | **P** |  |  |  |  |  |  |  |  | **P** | **N** | **P** | **P** |
| **Social IPV** |  | **P** | **N** |  | **P** |  |  |  | **N** |  |  |  |  | **P** |  |  |  |
| **Screened for IPV** |  |  | **P** | **N** |  |  |  |  | **P** |  |  |  |  |  | **P** |  |  |
| **Received information** |  |  |  |  | **N** |  |  |  |  |  |  |  |  |  |  |  |  |
| **Both screened and received information** |  |  |  | **N** | **N** |  |  |  |  |  |  |  |  |  |  | **N** | **N** |

^a^ 1. Family physician; 2. Gynecologist; 3. Specialist; 4. emergency room; and 5. hospitalization.

^b^N=Negative association, P=positive association, Otherwise- non-significant association.
