# Supplementary material for: Patterns of healthcare services utilization associated with intimate partner violence (IPV): Effects of IPV screening and receiving information on support services in a cohort of perinatal women
Source: PLoS One. 2020 Jan 31;15(1):e0228088. doi: 10.1371/journal.pone.0228088 (PMC6994004; doi:10.1371/journal.pone.0228088)
Supplement: S3 Appendix — (DOCX) [file pone.0228088.s003.docx]

**Appendix 3- Correlations between study variables**

|  | **1** | **2** | **3** | **4** | **5** | **6** | **7** |
| --- | --- | --- | --- | --- | --- | --- | --- |
| **1. Age** | 1.00 | 0.037 | .254^**^ | -.357^**^ | .427^**^ | .113^**^ | -0.060 |
| **2. Marital status** |  | 1.00 | -.092^**^ | -.121^**^ | -0.050 | .083^*^ | -.070^*^ |
| **3. Women’s education** |  |  | 1.00 | -.315^**^ | -0.024 | .213^**^ | -.089^*^ |
| **4. Ethnicity** |  |  |  | 1.00 | -0.021 | -.267^**^ | .187^**^ |
| **5. Mother of children** |  |  |  |  | 1.00 | .171^**^ | 0.043 |
| **6. Pregnancy status** |  |  |  |  |  | 1.00 | -0.032 |
| **7. Chronic illness** |  |  |  |  |  |  | 1.00 |
